# Fractional Lotka-Volterra-Type Cooperation Models: Impulsive Control on Their Stability Behavior

**DOI:** 10.3390/e22090970

**Published:** 2020-08-31

**Authors:** Rohisha Tuladhar, Fidel Santamaria, Ivanka Stamova

**Affiliations:** 1 Department of Biology, University of Texas at San Antonio, San Antonio, TX 78249, USA; rohisha.tuladhar@utsa.edu (R.T.); fidel.santamaria@utsa.edu (F.S.); 2 Department of Mathematics, University of Texas at San Antonio, San Antonio, TX 78249, USA

**Keywords:** biological Lotka-Volterra cooperation networks, fractional derivatives, impulsive control, Mittag-Leffler stability, practical stability, stability of sets

## Abstract

We present a biological fractional *n*-species delayed cooperation model of Lotka-Volterra type. The considered fractional derivatives are in the Caputo sense. Impulsive control strategies are applied for several stability properties of the states, namely Mittag-Leffler stability, practical stability and stability with respect to sets. The proposed results extend the existing stability results for integer-order n−species delayed Lotka-Volterra cooperation models to the fractional-order case under impulsive control.

## 1. Introduction

The dynamics of Lotka-Volterra and related systems are traditionally applied to problems in population dynamics [1,2,3,4]. For example, the work in Reference [5] studied a delayed cooperation ecosystem composed of a number (*n*) of species using the following Lotka-Volterra model:

(1) x˙i(t)=ri(t)xi(t)1−xi(t−τii)ai(t)+∑j=1j≠inbj(t)xj(t−τij)−ci(t)xi(t−τij),

where i,j=1,…,n; t≥0, $x_{i}\left(t\right)$ represents the density of the species *i* at time *t*, $r_{i}\left(t\right)$ are the intrinsic growth rates at time *t*, $a_{i}\left(t\right),$$b_{i}\left(t\right)$ and $c_{i}\left(t\right)$ are the cooperative coefficients, $\tau_{ii}$ denote the time delay due to gestation period of species *i*, $\tau_{ij}$, j≠i denote time delays related to the maturation of the species *i* and *j*, respectively, $0\leq \tau_{ij}\leq \tau$, τ=const, (i=1,2,…,n). It is assumed that only adult individuals of species *i* can benefit the species *j* and vice versa.

The model (1) has been originally introduced in Reference [6] for n=2 and $\tau_{ij}=0$, i,j=1,2, to describe the cooperative interaction between two species. Since then, it has been developed by several authors. See, for example, References [7,8,9].

An important objective in understanding biological systems is to determine their approach to equilibrium. Relative entropy, part of an information-theoretic approach, can be used to determine approach to equilibrium in biological systems, including the Lotka-Volterra type [10]. In addition, entropy methods are increasingly applied to biological models as robustness markers [11], as well as to determine information content in artificial neural networks [12].

Research on impulsive Lotka-Volterra and related systems has also increased because of their application to the study of population densities that are affected by some impulsive factors that cannot be ignored. Impulsive control strategies are also widely used in control theory of biological models [13,14,15,16,17,18,19,20]. In Reference [21] the author extended model (1) to the impulsive case and proved that the population dynamics of the system can be impulsively controlled. Furthermore, appropriate exterior or interior short-term changes in population densities may be used as controls. In general, impulsive controllers and impulsive control techniques demonstrate their efficiency in stability and synchronization of numerous applied models [22,23,24].

Fractional-order derivatives are increasingly being used to better reflect long-term dependence properties in biological systems [25,26,27,28]. Fractional Lotka-Voltera-type models have also been recently developed [29,30,31,32,33,34]. Indeed, the use of fractional derivatives and integrals has proven advantages in many applications across engineering and sciences [35,36,37]. In addition, the extended impulsive fractional-order case has also attracted the attention of many researchers [38,39,40]. Impulsive effects in fractional biological models are considered by several authors [38,39,41,42,43], including some fractional Lotka-Volterra models [39,44]. However, most of the studies considered competitive models, and to the best of our knowledge, there are no results on fractional cooperative models.

The asymptotic stability of equilibrium points is one of the most important tasks in the study of biological network systems. Asymptotic stability theory has been applied to a wide range of integer-order Lotka-Volterra systems to explain the behavior of the system’s solutions using stabilization control law [1,2,3,5,7,8,9,21,23]. For fractional-order models the concept of Mittag-Leffler stability, introduced in Reference [45], is adapted as a generalization of the exponential stability notion in integer-order systems [46,47,48]. Very recently, the Mittag-Leffler notion has been also applied to fractional-order control problems [49,50,51], including impulsive controls in neural network systems [47,52,53,54]. The Mittag-Leffler stability and impulsive control strategies have not been investigated for fractional generalizations of the cooperative Lotka-Volterra model (1) which are important and challenging problems and one of the main goals of the present research.

The concepts of practical stability [55,56] and stability of sets [57] have been applied to fractional-order systems [58,59,60,61]. Using a practical stability approaches is beneficial in the cases when the equilibrium of the considered system is unstable in the classical Lyapunov or Mittag-Leffler sense, but its performance may be sufficient for a particular application [56]. Stability of sets generalizes the concept of stability of a single solution and is suitable to numerous real-world applications [62,63]. However, due to the complexity of the problem, no results on practical stability and stability of sets properties have been reported so far for fractional Lotka-Volterra-type models.

Stimulated by the above discussion, in this paper, we study impulsive control strategies for the Mittag-Leffler stability, practical stability, and stability of sets behavior of the fractional-order modification of the n−species delayed cooperation Lotka-Volterra type model (1). Caputo’s fractional-order approach is adopted in our research. In Section 2 we will first introduce the fractional generalization of the biological network model (1). Some definitions and lemmas are also presented. Section 3 is devoted to our Mittag-Leffler stability results via impulsive controllers. In Section 4 the practical stability results for the fractional impulsive control biological model are derived. Section 5 generalizes the results in Section 3 and Section 4, and offers stability of sets results. In Section 6 examples and simulations are given. Section 7 presents our conclusions and future directions.

## 2. Fractional Lotka-Volterra-Type Model and Preliminary Notes

We will use the following notations: $R_{+}=\left[0,\infty \right)$, $R^{n}$ is the *n*-dimensional Euclidean space and $\left||x||=\right|x_{1}\left|+\ldots +\right|x_{n}|$ denote the norm of $x\in R^{n}$.

Let $u\in C^{1}\left[\left[0,b\right],R\right]$, b>0. For any 0<q<1, following Reference [36], we consider the Caputo fractional derivative of order *q* with the lower limit 0 for a function *u*, defined as

$${{}^{C}D}_{t}^{q}u\left(t\right)=\frac{1}{\Gamma (1-q)}\int_{0}^{t}\frac{u^{\prime }\left(\sigma \right)}{{\left(t-\sigma \right)}^{q}}d\sigma ,\;t\geq 0,$$

where Γ is the Gamma function and $u^{\prime }$ is the first-order ordinary derivative of the function *u*.

Since introducing fractional-order derivatives in population dynamics models has important practical significance and research values [25,26,27,28,29,30,31,32,33,34], we generalize the model (1), and introduce the following model with fractional-order derivatives

(2) CDtqxi(t)=ri(t)xi(t)1−xi(t−τii)ai(t)+∑j=1j≠inbj(t)xj(t−τij)−ci(t)xi(t−τij),

where i=1,2,…,n, n≥2, 0<q<1, the functions $r_{i}\left(t\right),\;a_{i}\left(t\right),\;b_{i}\left(t\right)$ and $c_{i}\left(t\right)$ are continuous, positive and bounded on $R_{+}$.

The fractional derivatives in the Lotka-Volterra-type cooperation model (2) will contribute for a better description of the population interactions characteristics and will improve the degree of freedom in comparison with the model (1) [35,36].

We will consider the following initial condition

(3) $$x_{i}\left(s;0,\varphi_{0}\right)=\varphi_{i0}\left(s\right),\;s\in \left[-\tau ,0\right]$$

associated with the model (2), where the initial function $\varphi_{0}:\left[-\tau ,0\right]\to R^{n}$, $\varphi_{0}={\left(\varphi_{10},\;\varphi_{20},\;\ldots ,\;\varphi_{n0}\right)}^{T}$ is continuous, positive and bounded on [−τ,0].

For a continuous function g(t) defined on [0,∞), we denote

$$g^{L}=\underset{t\in [0,\infty )}{\inf}g\left(t\right),\;g^{M}=\underset{t\in [0,\infty )}{\sup}g\left(t\right).$$

The goal of our research is to investigate the effects of impulsive external perturbations on the stability behavior of the model (2) at some fixed points $t_{1},t_{2},\ldots$ such that

$$0<t_{1}<t_{2}<\ldots <t_{k}<\ldots$$

and $\lim_{k\to \infty }t_{k}=\infty$. To this end we will add impulsive controllers that can be used to synchronize the densities of all species onto that of system (2), and introduce the following impulsive control system

(4) CDtqyi(t)=ri(t)yi(t)1−yi(t−τii)ai(t)+∑j=1j≠inbj(t)yj(t−τij)−ci(t)yi(t−τij)+ηi(yi(t)),

where

$$\eta_{i}\left(y_{i}\left(t\right)\right)=\sum_{k=1}^{\infty }I_{ik}\left(y_{i}\left(t\right)\right)\delta \left(t-t_{k}\right)$$

are the control inputs, δ(t) is the Dirac impulsive function, $y_{i}:R_{+}\to R_{+}$, $I_{ik}:R_{+}\to R$, i=1,…,n, k=1,2,⋯.

Due to the incorporation of the Delta impulsive function the controller η(t) has an effect on sudden changes in the states of the model (4) at the time instants $t_{k}$, that is, η(t) is an impulsive control of (4). For t>0 and i=1,2,…,n, we denote

fi(t,yi(t))=ri(t)yi(t)1−yi(t−τii)ai(t)+∑j=1j≠inbj(t)yj(t−τij)−ci(t)yi(t−τij).

From (4), we have that [35,36,39,61], $\eta_{i}\left(y_{i}\left(t\right)\right)=0$ for $t\neq t_{k},$k=1,2,…., i=1,2,…,n, and

$$y_{i}\left(t_{k}+h\right)-y_{i}\left(t_{k}\right)=\frac{1}{\Gamma (q)}\int_{0}^{t_{k}+h}{\left(t_{k}+h-\sigma \right)}^{q-1}\left[f_{i}\left(\sigma ,y_{i}\left(\sigma \right)\right)+\eta_{i}\left(y_{i}\left(\sigma \right)\right)\right]d\sigma$$

$$-\frac{1}{\Gamma (q)}\int_{0}^{t_{k}}{\left(t_{k}-\sigma \right)}^{q-1}\left[f_{i}\left(\sigma ,y_{i}\left(\sigma \right)\right)+\eta_{i}\left(y_{i}\left(\sigma \right)\right)\right]d\sigma$$

$$=\frac{1}{\Gamma (q)}\int_{t_{k}}^{t_{k}+h}{\left(t_{k}+h-\sigma \right)}^{q-1}\left[f_{i}\left(\sigma ,y_{i}\left(\sigma \right)\right)+\eta_{i}\left(y_{i}\left(\sigma \right)\right)\right]d\sigma$$

$$+\frac{1}{\Gamma (q)}\int_{0}^{t_{k}}\left({\left(t_{k}+h-\sigma \right)}^{q-1}-{\left(t_{k}-\sigma \right)}^{q-1}\right)\left[f_{i}\left(\sigma ,y_{i}\left(\sigma \right)\right)+\eta_{i}\left(\sigma \right)\right]d\sigma ,$$

where h>0 is sufficiently small. As $h\to 0^{+}$, we obtain

$$\Delta y_{i}\left(t_{k}\right)=y_{i}\left(t_{k}^{+}\right)-y_{i}\left(t_{k}\right)=I_{ik}\left(y_{i}\left(t_{k}\right)\right),$$

where $y_{i}\left(t_{k}^{+}\right)=\lim_{h\to 0^{+}}y_{i}\left(t_{k}+h\right)$.

Therefore, the correspondent *n*-dimensional fractional-order driven system is given by

(5) CDtqyi(t)=yi(t)ri(t)1−yi(t−τii)ai(t)+∑j=1j≠inbj(t)yj(t−τij)−ci(t)yi(t−τij),t≠tk,yi(tk+)=yi(tk)+Iik(yi(tk)),i=1,…,n,k=1,2,…,

where the quantities $y_{i}\left(t_{k}\right)$ and $y_{i}\left(t_{k}^{+}\right)=\lim_{h\to 0^{+}}y_{i}\left(t_{k}+h\right)$ represent, respectively, the population densities of species *i* before and after an impulsive perturbation at the moment $t_{k}$ and the continuous in $R_{+}$ functions $I_{ik}$ characterize the impact of the impulse effect on the species *i* at the moments $t_{k}$.

For more results on fractional-order models under impulsive control where the Dirac delta function is incorporated in a similar sense, we refer the reader to References [39,61] and the references therein.

Let $\phi_{0}:\left[-\tau ,0\right]\to R^{n}$, $\phi_{0}={\left(\phi_{10},\phi_{20},\ldots ,\phi_{n0}\right)}^{T}$ be a piecewise continuous function with points of discontinuity on [−τ,0] at which it is continuous from the left. The class of all such functions will be denoted by PC, and by $PCB[\left[-\tau ,0\right],R^{n}]$ we will denote the class of all bounded functions $\phi_{0}\in \mathrm{PC}$. We denote by $y\left(t\right)=y\left(t;0,\phi_{0}\right)={\left(y_{1}\left(t;0,\phi_{0}\right),y_{2}\left(t;0,\phi_{0}\right),\ldots ,y_{n}\left(t;0,\phi_{0}\right)\right)}^{T}$ the solution of system (5), satisfying the initial conditions

(6) $$\left\{\begin{array}{c} y_{i}\left(s;0,\phi_{0}\right)=\phi_{i0}\left(s\right),\;s\in \left[-\tau ,0\right],\\ y_{i}\left(0^{+};0,\phi_{0}\right)=\phi_{i0}\left(0\right),\;i=1,\ldots ,n. \end{array}\right.$$

Furthermore, from the biological point of view, we will restrict our attention only to the nonnegative solutions of the control system (5), and we will assume that if $\phi_{i0}\left(s\right)\geq 0$, $\sup\phi_{i0}\left(s\right)<\infty$, s∈[−τ,0], $\phi_{i0}\left(0\right)>0,$1≤i≤n, then $y_{i}\left(t\right)\geq 0$, 1≤i≤n, t∈[0,∞). We also assume that $y_{i}+I_{ik}\left(y_{i}\right)\geq 0$ for $y_{i}\in R_{+}$, i=1,2,…,n, k=1,2,….

For some results on positive solutions of fractional Lotka-Volterra-type models we refer to References [32,33].

In addition, we assume that, if the functions $I_{ik}$ are such that $-y_{i}\leq I_{ik}\left(y_{i}\right)\leq 0$ for $y_{i}\in R_{+}$, i=1,2,…,n, k=1,2,…, then there exist positive constants *m* and M<∞ such that

(7) $$m\leq y_{i}\left(t\right)\leq M,\;t\in \left[0,\infty \right).$$

For integer-order Lotka-Volterra models with and without impulses, similar results can be found in References [1,5,7,8,9,21,23]. Since one of the main advantages of Caputo’s fractional derivatives is that the initial conditions for fractional differential equations with such derivatives have a similar form as for integer-order differential equations, the validity of (7) can be verified using similar steps as in the integer-order models.

Define the norm ${\left||\phi |\right|}_{\tau }=\underset{s\in [-\tau ,0]}{\sup}\left||\phi \left(s\right)|\right|$ of the function $\phi \in \;PCB[\left[-\tau ,0\right],R^{n}].$

It is well known [1,2,4,7,8,9,13,21,23] that Lyapunov functions can be a successful instrument in the investigation of the stability properties of a Lotka-Volterra type system. To apply the Lyapunov function method, we introduce the following notations:

$G_{k}=\left(t_{k-1},t_{k}\right)\times R_{+}^{n},\;k=1,2,\ldots$; $G=\cup_{k=1}^{\infty }G_{k}$;

$V_{0}=\left\{V:\left[0,\infty \right)\times R_{+}^{n}\to R_{+}:V\in C\left[G,R_{+}\right],\;t\in \left[0,\infty \right)\right.$, *V* is locally Lipschitzian in $y\in R_{+}^{n}$ on each of the sets $G_{k}$, $V\left(t_{k}^{-},y\right)=V\left(t_{k},y\right)$ and V(tk+,y)=limt→tkt>tkV(t,y) exists}.

For $V\in V_{0}$ and $t\in [t_{k-1},t_{k})$, we will use the following fractional derivative of *V* of order *q*, 0<q<1 with respect to system (1) defined [39] by

$${}^{C}D_{+}^{q}V\left(t,\phi \left(0\right)\right)=\lim_{h\to 0^{+}}\sup\frac{1}{h^{q}}\left[V\left(t,\phi \left(0\right)\right)-V\left(t-h,\phi \left(0\right)-h^{q}f\left(t,\phi \right)\right)\right],$$

where $f\left(t,\phi \right)={\left(f_{1}\left(t,\phi \right),f_{2}\left(t,\phi \right),\ldots ,f_{n}\left(t,\phi \right)\right)}^{T}$ denotes the right-hand side of (5), $\phi \in PCB[\left[-\tau ,0\right],R^{n}]$,

fi(t,ϕ)=ϕi(t)ri(t)1−ϕi(t−τii)ai(t)+∑j=1j≠inbj(t)ϕj(t−τij)−ci(t)ϕi(t−τij).

We also recall the following basic comparison result of fractional calculus from Reference [39].

**Lemma** **1.** *Assume that the function $V\in V_{0}$ is such that for $t\in R_{+}$, ϕ∈PC,*

$$V\left(t^{+},\phi \left(0\right)+\Delta \phi \right)\leq V\left(t,\phi \left(0\right)\right),\;\;t=t_{k},$$

*and for a constant μ>0 the inequality*

$${}^{C}D_{+}^{q}V\left(t,\phi \left(0\right)\right)\leq -\mu V\left(t,\phi \left(0\right)\right),\;t\neq t_{k},\;k=1,2,\ldots$$

*is valid whenever V(t+s,ϕ(s))≤V(t,ϕ(0)) for −τ≤s≤0.*

*Then $sup_{-\tau \leq s\leq 0}V\left(s,\phi_{0}\left(s\right)\right)\leq V\left(t,\phi \left(0\right)\right)$ implies*

$$V\left(t,y\left(t;0,\phi_{0}\right)\right)\leq \underset{-\tau \leq s\leq 0}{\sup}V\left(0^{+},\phi_{0}\left(s\right)\right)E_{q}\left(-\mu t^{q}\right),\;t\in \left[0,\infty \right),$$

*where $E_{q}$ is the Mittag-Leffler function defined as $E_{q}\left(z\right)=\sum_{\kappa =0}^{\infty }\frac{z^{\kappa }}{\Gamma (q\kappa +1)},\;q>0$.*

For more comparison results and properties on Mittag-Leffler functions we refer to References [35,36,37,39].

## 3. Mittag-Leffler Stability Results

Let $\phi_{0}^{*}\in PCB\left[\left[-\tau ,0\right],R_{+}^{n}\right]$, $\phi_{0}^{*}={\left(\phi_{10}^{*},\;\phi_{20}^{*},\;\ldots ,\;\phi_{n0}^{*}\right)}^{T}$ and $y^{*}\left(t\right)=y^{*}\left(t;0,\phi_{0}^{*}\right)=$ $(y_{1}^{*}\left(t;0,\phi_{0}^{*}\right),$ $y_{2}^{*}\left(t;0,\phi_{0}^{*}\right),\ldots ,y_{n}^{*}\left(t;0,\phi_{0}^{*}\right){)}^{T}$ be the solution of the impulsive control system (5) with initial function $\phi_{0}^{*}$. In the next, we shall suppose that $\phi_{i0}^{*}\left(s\right)\geq 0$, $\sup\phi_{i0}^{*}\left(s\right)<\infty$, $\phi_{i0}^{*}\left(0\right)>0,\;i=1,2,\ldots ,n.$

We will use the following definition for Mittag-Leffler stability of the state $y^{*}\left(t\right)$ of the model (5) [45,46,47,48,49,50,51,52,53,54].

**Definition** **1.** The state $y^{*}\left(t\right)$ of system (5) is said to be globally Mittag-Leffler stable, if

$$||y\left(t;0,\phi_{0}\right)-y^{*}\left(t;0,\phi_{0}^{*}\right)\left|\right|\leq \left\{m[|\right|\phi_{0}-\phi_{0}^{*}{\left|\right|}_{\tau }{]E_{q}\left(-\mu t^{q}\right)\}}^{d},$$

*where $E_{q}$ is the corresponding Mittag-Leffler function, q∈(0,1), μ>0, d>0, m(0)=0, m(ϕ)≥0, and m(ϕ) is locally Lipschitz with respect to $\phi \in PCB[\left[-\tau ,0\right],R^{n}]$.*

In some of the literature [50], the constant μ is also called the degree of Mittag-Leffler state estimator, which can be considered as an equivalence of the convergence rate as state estimator error tends to zero when time *t* goes to infinity.

Our main result in this section is the next theorem.

**Theorem** **1.** *Assume that:*

*1.*  *The model’s parameters satisfy*
  2mmin1≤i≤nriLaiM+M∑j=1j≠inbjM+min1≤i≤nriLciL>max1≤i≤nriM+max1≤i≤n∑j=1j≠inbjMriMM2aiL+m∑κ=1κ≠jnbκL2.
*2.*  *The impulsive functions $I_{ik}$ are such that*
  (8) $$I_{ik}\left(y_{i}\left(t_{k}\right)\right)=-\gamma_{ik}\left(y_{i}\left(t_{k}\right)-y_{ik}^{*}\left(t_{k}\right)\right),\;0<\gamma_{ik}<2,\;i=1,2,\ldots n,\;k=1,2,\ldots .$$
Then, the state $y^{*}\left(t\right)$ of system (5) is globally Mittag-Leffler stable.

**Proof.**  The proof is based on the Lyapunov-Razumikhin technique [39]. We consider the following Lyapunov function candidate

$$V\left(t,y\right)=\sum_{i=1}^{n}\left|y_{i}-y_{i}^{*}\right|.$$

For k=1,…, and ϕ∈PCB, according to (8), we have

(9) $$\begin{array}{c} V\left(t_{k}^{+},\phi \left(0\right)+I_{k}\left(\phi \right)\right)=\sum_{i=1}^{n}\left|\phi_{i0}\left(0\right)+I_{ik}\left(\phi_{0}\right)-\phi_{i0}^{*}\left(0\right)-I_{ik}\left(\phi_{0}^{*}\right)\right|\\ \leq \sum_{i=1}^{n}\left|\left(1-\gamma_{ik}\right)\left(\phi_{i0}\left(0\right)-\phi_{i0}^{*}\left(0\right)\right)\right|\leq V\left(t_{k},\phi \left(0\right)\right). \end{array}$$

Also, for t≥0, $t\neq t_{k}$, k=1,2,…, and ϕ∈PCB, we estimate the derivative ${}^{C}D_{+}^{q}V\left(t,\phi \left(0\right)\right)$ along the system (5), and get that

(10) CD+qV(t,ϕ(0))≤max1≤i≤nriM∑i=1n|ϕi0(0)−ϕi0*(0)|−min1≤i≤n2riLciLm∑i=1n|ϕi0(0)−ϕi0*(0)|−min1≤i≤n2riLmaiM+M∑j=1j≠inbjM∑i=1n|ϕi0(0)−ϕi0*(0)|+max1≤i≤n∑j=1j≠inbjMriMM2aiL+m∑κ=1κ≠jnbκL2∑i=1nsups∈[−τ,0]|ϕi0(s)−ϕi0*(s)|≤(−2mmin1≤i≤nriLaiM+M∑j=1j≠inbjM+min1≤i≤nriLciL+max1≤i≤nriM+max1≤i≤n∑j=1j≠inbjMriMM2aiL+m∑κ=1κ≠jnbκL2)V(t,φ(0))

is valid whenever V(t+s,φ(s))≤V(t,φ(0)) for −τ≤s≤0.

In view of (10) and condition 1 of Theorem 1, there exists a positive μ such that

(11) $${}^{C}D_{+}^{q}V\left(t,\phi \left(0\right)\right)\leq -\mu V\left(t,\phi \left(0\right)\right),\;t\neq t_{k},\;k=1,2,\ldots ,$$

whenever V(t+s,ϕ(s))≤V(t,ϕ(0)) for −τ≤s≤0.

From (9) and (11), by Lemma 1, it follows that

$$V\left(t,y\left(t;0,\phi_{0}\right)\right)\leq \underset{-\tau \leq s\leq 0}{\sup}V\left(0^{+},\phi_{0}\left(s\right)\right)E_{q}\left(-\mu t^{q}\right),\;t\in \left[0,\infty \right).$$

So,

$$||y\left(t\right)-y^{*}\left(t\right)||\leq \left|\right|\phi_{0}-\phi_{0}^{*}{\left|\right|}_{\tau }E_{q}\left(-\mu t^{q}\right),\;t\in \left[0,\infty \right),$$

and this completes the proof of the theorem. □

**Remark** **1.** *The assumptions of Theorem 1 guarantee the Mittag-Leffler stability of the states of the impulsive control system (5). The impulsive control strategy is designed by (8). Therefore, the proposed structure of the control law can be applied to design impulsive based observers to estimate the states of (5) and globally Mittag-Leffler synchronize the states of the system (5) onto that of system (2). Note that, the synchronization scheme is independent on the lengths of the impulsive intervals.*

**Remark** **2.** *One of the states that is of a great interest to researchers of Lotka-Volterra and related models are the so called “steady" or equilibrium states [1,3,21,47]. Let*

$$y^{*}={\left(y_{1}^{*},y_{2}^{*},\ldots ,y_{n}^{*}\right)}^{T}$$

*be the equilibrium of the control system (5) such that*

1=yi*ai(t)+∑j=1j≠inbj(t)yj*−ci(t)yi*,t≠tk,

$$I_{ik}\left(y_{i}^{*}\right)=0,\;\;\;i=1,2,\ldots ,n,\;\;k=1,2,\ldots .$$

Since $I_{ik}\left(y_{i}^{*}\right)=0,\;\;\;i=1,2,\ldots ,n,\;\;k=1,2,\ldots$, the state $y^{*}$ is also a steady state for the controlled model (2). Hence, in the case when $y^{*}\left(t\right)=y^{*}=const$, the providing results in Theorem 1 can be used so by means of convenient impulsive control strategy to keep the Mittag-Leffler stability properties of the equilibrium states of system (2). Note that the Mittag-Leffler stability for fractional-order models corresponds to the exponential stability for integer-order systems, which guarantees the fast convergence rate.

## 4. Practical Stability Results

In this section, following Reference [56] we will provide practical stability results for the impulsive control model (5).

We introduce the following definition.

**Definition** **2.** The state $y^{*}\left(t\right)$ of the fractional impulsive control system (5) is said to be:

(a) practically uniformly globally stable with respect to (λ,A), if given (λ,A) with 0<λ<A, we have $\left|\right|\phi_{0}-\phi_{0}^{*}{\left|\right|}_{\tau }<\lambda$ implies

$$||y\left(t;0,\phi_{0}\right)-y^{*}\left(t;0,\phi_{0}^{*}\right)\left|\right|\leq A,\;t\geq 0;$$

(b) practically uniformly globally Mittag-Leffler stable with respect to (λ,A), if given (λ,A) with 0<λ<A, we have $\left|\right|\phi_{0}-\phi_{0}^{*}{\left|\right|}_{\tau }<\lambda$ implies

$$||y\left(t;0,\phi_{0}\right)-y^{*}\left(t;0,\phi_{0}^{*}\right)\left|\right|\leq A+\nu ||\phi_{0}-\phi_{0}^{*}{\left|\right|}_{\tau }E_{q}\left[-\mu t^{q}\right],\;t\geq 0,\;\nu ,\mu >0.$$

Condition 1 of Theorem 1 guarantees the existence of a positive constant μ such that (11) holds. The application of (11) together with the appropriate choice of the Lyapunov-candidate function *V* imply the global Mittag-Leffler stability of the state $y^{*}\left(t\right)$ of the control system (5) by means of the designed impulsive control law (8). This section will firstly address the case, when μ=0 and the state $y^{*}\left(t\right)$ is not globally Mittag-Leffler stable. We will prove that in such case the impulsive controller (8) may assure its practical uniform global stability.

We will use again the Lyapunov function candidate

$$V\left(t,y\right)=\sum_{i=1}^{n}\left|y_{i}-y_{i}^{*}\right|$$

and functions of the following class:

$K=\{a\in C[R_{+},R_{+}]:\;a\left(u\right)$ is strictly increasing in *u* and a(0)=0}.

**Theorem** **2.** *Assume that the impulsive controller satisfies (8) and:*

*1. 0<λ<A are given.*

*2. The model’s parameters satisfy*

2mmin1≤i≤nriLaiM+M∑j=1j≠inbjM+min1≤i≤nriLciL=max1≤i≤nriM+max1≤i≤n∑j=1j≠inbjMriMM2aiL+m∑κ=1κ≠jnbκL2.

*3. There exists a function b∈K such that*

$$V\left(t,y\right)\leq b(||y-y^{*}||),\;t\in R_{+},\;y,y^{*}\in R^{n}.$$

*4. b(λ)<A holds.*

Then, the state $y^{*}\left(t\right)$ of system (5) is practically uniformly globally stable with respect to (λ,A).

**Proof.**  Let 0<λ<A. We will prove that $\left|\right|\phi_{0}-\phi_{0}^{*}{\left|\right|}_{\tau }<\lambda$ implies $||y\left(t;0,\phi_{0}\right)-y^{*}\left(t;0,\phi_{0}^{*}\right)\left|\right|\leq A,\;t\geq 0$.

If this is not true, there exists a corresponding solution $y(t;0,\phi_{0})$ of (5) with $\left|\right|\phi_{0}-\phi_{0}^{*}{\left|\right|}_{\tau }<\lambda$, and $0<t^{*}<\infty$, $t_{k}<t^{*}\leq t_{k+1}$ for some *k*, such that

$$||y\left(t;0,\phi_{0}\right)-y^{*}\left(t;0,\phi_{0}^{*}\right)||<A,\;t\in \left[0,t_{k}\right]\;\;\;\mathrm{and}\;\;||y\left(t^{*}\right)-y^{*}\left(t^{*}\right)\left|\right|\geq A.$$

Note that the impulsive control low (8) guarantees that if

$$||y\left(t_{k};0,\phi_{0}\right)-y^{*}\left(t_{k};0,\phi_{0}^{*}\right)\left|\right|<A,$$

then

$$||y\left(t_{k}^{+};0,\phi_{0}\right)-y^{*}\left(t_{k}^{+};0,\phi_{0}^{*}\right)\left|\right|=\sum_{i=1}^{n}\left|y_{i}\left(t_{k}\right)+I_{ik}\left(y_{i}\left(t_{k}\right)\right)-y_{i}^{*}\left(t_{k}\right)-I_{ik}\left(y_{i}^{*}\left(t_{k}\right)\right)\right|$$

$$\leq \sum_{i=1}^{n}\left|\left(1-\gamma_{ik}\right)\left(y_{i}\left(t_{k}\right)-y_{i}^{*}\left(t_{k}\right)\right)\right|<A.$$

Hence $t^{*}\neq t_{k}$ for any k=1,2,….

From condition 2 of Theorem 2 by similar arguments as in the proof of Theorem 1, we have

(12) $${}^{C}D_{+}^{q}V\left(t,\phi \left(0\right)\right)\leq 0,\;t\neq t_{k},\;k=1,2,\ldots ,$$

whenever V(t+s,ϕ(s))≤V(t,ϕ(0)) for −τ≤s≤0.

Also, using (12) and (8), we obtain by Lemma 1, for μ=0

(13) $$V\left(t,y\left(t;0,\phi_{0}\right)\right)\leq \underset{-\tau \leq s\leq 0}{\sup}V\left(0^{+},\phi_{0}\left(s\right)\right),\;t\in \left[0,t^{*}\right].$$

Hence, (13), (8) and condition 4 of Theorem 2 imply

$$A\leq V\left(t^{*},y\left(t^{*};0,\phi_{0}\right)\right)\leq \underset{-\tau \leq s\leq 0}{\sup}V\left(0^{+},\phi_{0}\left(s\right)\right)\leq b\left(\lambda \right)<A.$$

The contradiction obtained proves our claim that system (5) is practically uniformly globally stable with respect to (λ,A). □

**Remark** **3.** In Theorem 2 we proved that even in some concrete cases, when the system (5) is not globally Mittag-Leffler stable, by the impulsive controller (8) we can made the vicinity of a state $y^{*}$ arbitrarily small so that all other states to remain arbitrarily close to it provided that their initial functions were sufficiently near the initial data of the particular state $y^{*}$. The property of practical stability is very important in many applications when the Mittag-Leffler stability is conservative [58,60,61]. For type (2) models from the population biology this can compensate a not stable initial population by keeping the population densities between particular bounds for t≥0.

**Theorem** **3.** *Assume that:*

*1. There exists a function $V\in V_{0}$ for which conditions of Lemma 1 are met.*

*2. The function V(t,y) is such that*

$$||y-y^{*}\left|\right|-A\leq V\left(t,y\right)\leq a\left(u\right)||y-y^{*}\left|\right|,\;t\in R_{+},\;y,y^{*}\in R^{n},$$

*where the continuous function a(u)≥1 exists for any u>0.*

*3. Condition 1 of Theorem 2 holds.*

Then, the state $y^{*}\left(t\right)$ of system (5) is practically uniformly globally Mittag-Leffler stable with respect to (λ,A).

**Proof.**  Let 0<λ<A, and $\left|\right|\phi_{0}-\phi_{0}^{*}{\left|\right|}_{\tau }<\lambda$ for an initial function $\phi_{0}\in PCB\left[\left[-\tau ,0\right],R^{n}\right]$ of an arbitrary solution $y\left(t\right)=y\left(t;0,\phi_{0}\right)$ of the initial value problem (5), (6).

From Lemma 1, we have that

(14) $$V\left(t,y\left(t;0,\phi_{0}\right)\right)\leq \underset{-\tau \leq s\leq 0}{\sup}V\left(0^{+},\phi_{0}\left(s\right)\right)E_{q}\left(-\mu t^{q}\right),\;t\in \left[0,\infty \right),$$

where μ>0.

From (14) and condition 2 of Theorem 3, we have

$$||y\left(t;0,\phi_{0}\right)-y^{*}\left(t;0,\phi_{0}^{*}\right)\left|\right|-A\leq V\left(t,y\left(t;0,\phi_{0}\right)\right)$$

$$\leq \underset{-\tau \leq s\leq 0}{\sup}V\left(0^{+},\phi_{0}\left(s\right)\right)E_{q}\left(-\mu t^{q}\right)\leq a\left(u\right)\left|\right|\phi_{0}-\phi_{0}^{*}{\left|\right|}_{\tau }E_{q}\left(-\mu t^{q}\right),\;\;\;t\geq 0.$$

Therefore, for any ν≥a(u), u>0, we have

$$||y\left(t;0,\phi_{0}\right)-y^{*}\left(t;0,\phi_{0}^{*}\right)\left|\right|\leq A+\nu ||\phi_{0}-\phi_{0}^{*}{\left|\right|}_{\tau }E_{q}\left(-\mu t^{q}\right),\;\;\;t\geq 0,$$

which shows that system (5) is practically uniformly globally Mittag-Leffler stable with respect to (λ,A). □

**Remark** **4.** *For A=0 Theorem 3 implies global Mittag-Leffler stability. However, Theorem 3 can be applied when this stability notion is pointless from the applied point of view. In such cases the designed impulsive controller allows the practical uniform global Mittag-Leffler stability.*

**Remark** **5.** *More detailed explanations about the importance of the practical stability notion for applied problems, as well as, for relations between stability and practical stability concepts can be found in References [56,61,64].*

## 5. Stability of Sets

In this Section, we extend the Lyapunov function method to stability of sets criteria for the impulsive control system (5). In fact, considering closed sets of states as attractors instead of equilibrium states is common in population dynamics [1].

Let $B\subset R^{n}$ be an arbitrary set (compact or not compact). Define the norms:

${\left||y|\right|}^{B}=\inf\left\{||y-z||:\;z\in B\right\}$ is the distance from $y\in R^{n}$ to *B*;

${\left||\phi |\right|}_{\tau }^{B}=\underset{s\in [-\tau ,0]}{\sup}{\left||\phi \left(s\right)|\right|}^{B}$ is the distance between a function $\phi \in PCB[\left[-\tau ,0\right],R^{n}]$ and *B*.

**Definition** **3.** *The solutions of system (5) are said to be uniformly bounded with respect to the set B, if for any positive constant η>0 there exists a constant β>0 such that $\phi_{0}\in PCB\left[\left[-\tau ,0\right],R^{n}\right]$ and $\left|\right|\phi_{0}{\left|\right|}_{\tau }^{B}\leq \eta$ imply*

$$||y\left(t;0,\phi_{0}\right){\left|\right|}^{B}\leq \beta ,\;t\geq 0.$$

**Definition** **4.** *The set B is said to be globally Mittag-Leffler stable with respect to system (5), if the solutions of system (5) are uniformly bounded with respect to the set B, and*

$$||y\left(t;0,\phi_{0}\right){\left|\right|}^{B}\leq \left\{m[|\right|\phi_{0}{\left|\right|}_{\tau }^{B}{]E_{q}\left(-\mu t^{q}\right)\}}^{d},$$

*where $E_{q}$ is the corresponding Mittag-Leffler function, q∈(0,1), μ>0, d>0, m(0)=0, m(ϕ)≥0, and m(ϕ) is locally Lipschitz with respect to $\phi \in PCB[\left[-\tau ,0\right],R^{n}]$.*

**Remark** **6.** The notion of stability of sets with respect to system (5) generalizes the stability of single states concepts of system (5). For example, when $B=\{y\in R^{n}:\;y=y^{*}\left(t\right),\;t\geq 0\}$, Definition 3 reduces to Definition 1 for the global Mittag-Leffler stability of the state $y^{*}\left(t\right)$, t≥0.

**Remark** **7.** *The requirement for uniform boundedness of all solutions of (5) with respect to B is required by the fact that for noncompact sets B there is an opportunity that some solutions may tend to infinity in finite time within the set. If the set B is compact (for example, the equilibrium state), then the boundedness condition is surplus.*

**Theorem** **4.** *Assume that:*

*1. There exists a function $V\in V_{0}$ such that V(t,y)=0 for y∈B and V(t,y)>0 for $y\in R_{+}^{n}\backslash B$, t≥0.*

*2. There exists a function b∈K such that*

$${\left||y|\right|}^{B}\leq V\left(t,y\right)\leq {b(||y||}^{B}),\;\left(t,y\right)\in R_{+}\times R_{+}^{n}.$$

*3. For the function V conditions of Lemma 1 are met.*

*Then, the set B is globally Mittag-Leffler stable with respect to system (5).*

**Proof.**  We will first prove the uniform boundedness of the solutions of (5) with respect to the set *B*. Let η>0 be given and let $\phi_{0}\in PCB\left[\left[-\tau ,0\right],R^{n}\right]$ be such that $\left|\right|\phi_{0}{\left|\right|}_{\tau }^{B}\leq \eta$. We can choose β>0 so that b(η)<β.

Let $y(t;0,\phi_{0})$ be the solution of the system (5) corresponding to the initial function $\phi_{0}$.

Since the conditions of Lemma 1 are met, then for μ=0, we have

(15) $$V\left(t,y\left(t;0,\phi_{0}\right)\right)\leq \underset{-\tau \leq s\leq 0}{\sup}V\left(0^{+},\phi_{0}\left(s\right)\right),\;t\geq 0.$$

Now, condition 2 of Theorem 4 and (15) imply the inequalities

$$||y\left(t;0,\phi_{0}\right){\left|\right|}^{B}\leq V\left(t,y\left(t;0,\phi_{0}\right)\right)\leq \underset{-\tau \leq s\leq 0}{\sup}V\left(0^{+},\phi_{0}\left(s\right)\right)$$

$$\leq b(||\phi_{0}|{|}_{\tau }^{B})\leq b\left(\eta \right)<\beta ,\;t\geq 0.$$

Therefore,

$$||y\left(t;0,\phi_{0}\right){\left|\right|}^{B}\leq \beta ,\;t\geq 0.$$

Since the solution $y(t;0,\phi_{0})$ is arbitrary the above proves the uniform boundedness of the solutions of (5) with respect to the set *B*.

In a similar way, as above, using Lemma 1 and condition 2 of Theorem 4, we obtain

$$||y\left(t;0,\phi_{0}\right){\left|\right|}^{B}\leq V\left(t,y\left(t;0,\phi_{0}\right)\right)\leq \underset{-\tau \leq s\leq 0}{\sup}V\left(0^{+},\phi_{0}\left(s\right)\right)E_{q}\left(\mu t^{q}\right)$$

$$\leq b(||\phi_{0}{\left|\right|}_{\tau }^{B})E_{q}\left(\mu t^{q}\right),\;t\geq 0,$$

which according to Definition 4 means that the set B is globally Mittag-Leffler stable set with respect to (5). □

**Remark** **8.** *Theorem 4 shows that the impulsive control law (8) is designed so that gets global Mittag-Leffler stability of a set B with respect to the fractional model (5). For the integer-order systems this is equivalent to the fact that all trajectories arrive at a given target set B. Hence, the proposed result is of a great importance in the cases when the information on the system’s parameters is incomplete and ones has to conduct a robust stability analysis.*

**Remark** **9.** *It is well know that if B is globally asymptotically stable with respect to (5), then the trivial solution of (5) is practically stable [65].*

**Remark** **10.** *We can also define the hybrid concept of practical stability of an arbitrary set B with respect to the impulsive control system (5). In fact, this concept is more natural than that of a neighbourhood of a state. In this case, one important question will be how well the stability behavior can be impulsively controlled, which is a subject for our future research.*

## 6. Examples and Simulations

**Example** **1.** *Consider the following two-species fractional cooperation Lotka-Volterra system*

(16) $$\left\{\begin{array}{c} {{}^{C}D}_{t}^{q}x_{1}\left(t\right)=x_{1}\left(t\right)\left[1-\frac{x_{1}\left(t-\tau_{11}\right)}{1+5x_{2}\left(t-\tau_{12}\right)}-3x_{1}\left(t-\tau_{12}\right)\right],\\ \;{{}^{C}D}_{t}^{q}x_{2}\left(t\right)=x_{2}\left(t\right)\left[1-\frac{x_{2}\left(t-\tau_{22}\right)}{0.3+0.7x_{1}\left(t-\tau_{21}\right)}-3x_{2}\left(t-\tau_{21}\right)\right], \end{array}\right.$$

*t≥0, $0\leq \tau_{ij}\leq 1$, i,j=1,2.*

*We can easily check that the point $\left(\frac{2}{7},\frac{1}{5}\right)$ is an equilibrium for the model (16).*

*The corresponding fractional control Lotka-Volterra system is given by*

(17) $$\left\{\begin{array}{c} {{}^{C}D}_{t}^{q}y_{1}\left(t\right)=y_{1}\left(t\right)\left[1-\frac{y_{1}\left(t-\tau_{11}\right)}{1+5y_{2}\left(t-\tau_{12}\right)}-3y_{1}\left(t-\tau_{12}\right)\right],\;t\neq t_{k},\\ \;{{}^{C}D}_{t}^{q}y_{2}\left(t\right)=y_{2}\left(t\right)\left[1-\frac{y_{2}\left(t-\tau_{22}\right)}{0.3+0.7y_{1}\left(t-\tau_{21}\right)}-3y_{2}\left(t-\tau_{21}\right)\right],\;t\neq t_{k}, \end{array}\right.$$

*under impulsive control law*

(18) $$\left\{\begin{array}{c} \Delta y_{1}\left(t_{k}\right)=y_{1}\left(t_{k}^{+}\right)-y_{1}\left(t_{k}\right)=-\frac{2}{5}\left(y_{1}\left(t_{k}\right)-\frac{2}{7}\right),\;k=1,2,\ldots ,\\ \;\Delta y_{2}\left(t_{k}\right)=y_{2}\left(t_{k}^{+}\right)-y_{2}\left(t_{k}\right)=-\frac{1}{3}\left(y_{2}\left(t_{k}\right)-\frac{1}{5}\right),\;k=1,2,\ldots , \end{array}\right.$$

*where $0<t_{1}<t_{2}<\ldots$ and $\lim_{k\to \infty }t_{k}=\infty$.*

We have that for $y_{1}^{*}=\frac{2}{7}$ and $y_{2}^{*}=\frac{1}{5}$, ${{}^{C}D}_{t}^{q}y_{1}^{*}=0$ and ${{}^{C}D}_{t}^{q}y_{2}^{*}=0$, that is, the state $\left(\frac{2}{7},\frac{1}{5}\right)$ is an equilibrium for the model (17), (18) too.

*Also, obviously all conditions of Theorem 1 are satisfied for $m=\frac{1}{5}$, $M=\frac{2}{7}$, $a_{1}^{L}=a_{1}^{M}=1$, $a_{2}^{L}=a_{2}^{M}=0.3$, $b_{1}^{L}=b_{1}^{M}=0.7$, $b_{2}^{L}=b_{2}^{M}=5$, $c_{i}^{L}=c_{i}^{M}=3$, i=1,2, $\gamma_{1k}=\frac{2}{5}$, $\gamma_{2k}=\frac{1}{3}$, k=1,2,…. In fact, we have that*

1.364≈2mmin1≤i≤2riLaiM+M∑j=1j≠inbjM+min1≤i≤2riLciL>max1≤i≤2riM+max1≤i≤2∑j=1j≠i2bjMriMM2aiL+m∑κ=1κ≠j2bκL2≈1.314.

**Figure 1:**
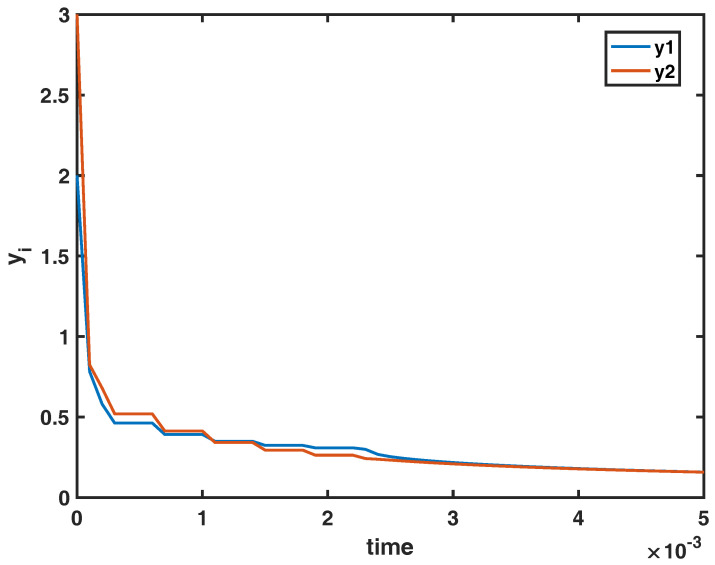
The global Mittag-Leffler stable behavior of the state variables $y_{1}\left(t\right)$ and $y_{2}\left(t\right)$ of the model (17) and (18).

*Hence, the equilibrium state $\left(\frac{2}{7},\frac{1}{5}\right)$ of the impulsive system (17), (18) is globally Mittag-Leffler stable. This example shows the effectiveness of the obtained results under an impulsive controller (18) of type (8). It also, demonstrates that the proposed sufficient conditions are reasonably strong. The global Mittag-Leffler stable behavior of the state variables $y_{1}\left(t\right)$ and $y_{2}\left(t\right)$ is demonstrated on Figure 1 for q=0.4, $x_{1}\left(s\right)=y_{1}\left(s\right)=2$, $x_{2}\left(s\right)=y_{2}\left(s\right)=3$, s∈[−0.01,0], $y_{1}\left(0^{+}\right)=2$, $y_{2}\left(0^{+}\right)=3$ and $t_{k}=\left[0.03,0.07,0.11,0.15,0.19,0.23\right]$.*

**Example** **2.** Consider again the fractional impulsive Lotka-Volterra system (17) and (18) with a globally Mittag-Leffler stable equilibrium state $y^{*}=\left(\frac{2}{7},\frac{1}{5}\right)$. Let the region of the Mittag-Leffler stability be determined by

$$||\phi -y^{*}{\left|\right|}_{\tau }<k.$$

*If λ and A are such that*

k<λ<A,

*then for an initial function $\phi_{0}={\left(\phi_{01},\phi_{02}\right)}^{T}$ such that*

$$k\leq ||\phi -y^{*}{\left|\right|}_{\tau }=\underset{s\in [-\tau ,0]}{\sup}||\phi \left(s\right)-y^{*}\left|\right|=\underset{s\in [-\tau ,0]}{\sup}\left(|\phi_{01}\left(s\right)-\frac{2}{7}|+|\phi_{02}\left(s\right)-\frac{1}{5}|\right)<\lambda$$

*the state $\left(\frac{2}{7},\frac{1}{5}\right)$ will not be practically uniformly globally Mittag-Leffler stable. Note that in the particular example, condition 2 of Theorem 2 is not satisfied.*

*The example again shows that Mittag-Leffler stability is not enough for practical Mittag-Leffler stability.*

**Example** **3.** *Consider the two-species fractional cooperation Lotka-Volterra model*

(19) $$\left\{\begin{array}{c} {{}^{C}D}_{t}^{q}x_{1}\left(t\right)=x_{1}\left(t\right)\left[1-\frac{x_{1}\left(t-\tau_{11}\right)}{1+5x_{2}\left(t-\tau_{12}\right)}-\frac{13}{4}x_{1}\left(t-\tau_{12}\right)\right],\\ \;{{}^{C}D}_{t}^{q}x_{2}\left(t\right)=x_{2}\left(t\right)\left[1-\frac{x_{2}\left(t-\tau_{22}\right)}{1+7x_{1}\left(t-\tau_{21}\right)}-\frac{4}{3}x_{2}\left(t-\tau_{21}\right)\right], \end{array}\right.$$

*t≥0, $0\leq \tau_{ij}\leq 1$, i,j=1,2.*

*We can easily check that the point $\left(\frac{2}{7},\frac{3}{5}\right)$ is an equilibrium for the model (19). However, since*

2mmin1≤i≤2riLaiM+M∑j=1j≠inbjM+min1≤i≤2riLciL<max1≤i≤2riM+max1≤i≤2∑j=1j≠i2bjMriMM2aiL+m∑κ=1κ≠j2bκL2

*we cannot make any conclusion about its global Mittag-Leffler stability.*

*For the corresponding fractional impulsive control Lotka-Volterra system*

(20) $$\left\{\begin{array}{c} {{}^{C}D}_{t}^{q}y_{1}\left(t\right)=y_{1}\left(t\right)\left[1-\frac{y_{1}\left(t-\tau_{11}\right)}{1+5y_{2}\left(t-\tau_{12}\right)}-\frac{13}{4}y_{1}\left(t-\tau_{12}\right)\right],\;t\neq t_{k},\\ \;{{}^{C}D}_{t}^{q}y_{2}\left(t\right)=y_{2}\left(t\right)\left[1-\frac{y_{2}\left(t-\tau_{22}\right)}{1+7y_{1}\left(t-\tau_{21}\right)}-\frac{4}{3}y_{2}\left(t-\tau_{21}\right)\right],\;t\neq t_{k},\\ \;\Delta y_{1}\left(t_{k}\right)=-\frac{2}{3}\left(y_{1}\left(t_{k}\right)-\frac{2}{7}\right),\;k=1,2,\ldots ,\\ \;\Delta y_{2}\left(t_{k}\right)=-\frac{3}{7}\left(y_{2}\left(t_{k}\right)-\frac{3}{5}\right),\;k=1,2,\ldots \end{array}\right.$$

*with $0<t_{1}<t_{2}<\ldots$, $\lim_{k\to \infty }t_{k}=\infty$ consider the Lyapunov function*

$$V\left(t,y\right)=\sum_{i=1}^{n}\left|y_{i}-y_{i}^{*}\right|.$$

**Figure 2:**
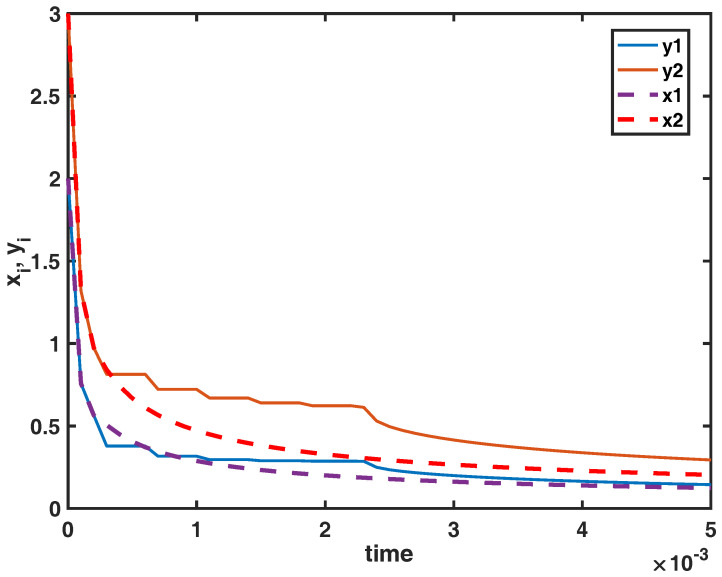
The practical uniform global Mittag-Leffler stable behavior of the model (20). The state variables $x_{1}\left(t\right)$ and $x_{2}\left(t\right)$ of (19) and $y_{1}\left(t\right)$ and $y_{2}\left(t\right)$ of (20).

*Let λ and A be given, and a(u)=u+1, $u\in R_{+}$. Then all conditions of Theorem 3 are satisfied, and hence, the equilibrium $\left(\frac{2}{7},\frac{3}{5}\right)$ of (20) is practically uniformly globally Mittag-Leffler stable, which means that the impulsive controllers contribute to keep the population densities of both spaces between given bounds. For example, in the case q=0.4, $t_{k}=\left[0.03,0.07,0.11,0.15,0.19,0.23\right]$, $\tau_{ij}=0.01,\;i,j=1,2$, $x_{1}\left(s\right)=y_{1}\left(s\right)=2$, $x_{2}\left(s\right)=y_{2}\left(s\right)=3,\;s\in \left[-0.01,0\right]$, $y_{1}\left(0^{+}\right)=2$, $y_{2}\left(0^{+}\right)=3$, the practical uniform global Mittag-Leffler stable behavior of the model (20) is shown in Figure 2.*

**Example** **4.** *For the fractional impulsive Lotka-Volterra model (17), (18) consider the set*

$$B=\{z\in R_{+}^{2}:\;0\leq z\leq y^{*}\},$$

where $y^{*}=\left(y_{1}^{*},y_{2}^{*}\right)=\left(\frac{2}{7},\frac{1}{5}\right)$.

*Following the steps in the proof of Theorem 1 using the Lyapunov function*

$$V\left(t,y\right)={\left||y|\right|}^{B}$$

*it is trivial to show that the set B is globally Mittag-Leffler stable with respect to (17), (18).*

## 7. Conclusions

In this paper, we introduce a fractional-order Lotka-Volterra type cooperation model. Based on Lyapunov function theory we present an impulsive control strategy that can be applied as a Mittag-Leffler stability and synchronization mechanism. In addition, more general practical stability results and stability of sets results with respect to the impulsive control system are established. Examples are presented to demonstrate the obtained criteria and the relations between the investigated stability behaviors. It is shown that the proposed impulsive control technique provides a useful tool for the design of appropriate population models. It can be also beneficial to models with reaction-diffusion terms which are the subject of our future research.
